# Integrative analysis of hybridization, metabolomic diversity, and ecological interactions in willow (*Salix*) species

**DOI:** 10.1093/jxb/erag125

**Published:** 2026-05-20

**Authors:** Henry Njoku

**Affiliations:** University of Ibadan, Ibadan, Nigeria

**Keywords:** Herbivore–plant interactions, hybridization, plant metabolomics, *Salix alba* × *fragilis;* structural chemical diversity

## Abstract

This article comments on:

**Renoult SA, Leong JV, Mezzomo P, Sebek P, Koutecký P, Kukla J, Nguyen P, Moos M, Pokorny V, Wagner ND, Frouz J, van Dam NM, Volf M**. 2025. Effects of hybridization on chemical diversity and plant–insect herbivore interactions in *Salix alba×fragilis*. Journal of Experimental Botany **76**, 6974–6986. https://doi.org/10.1093/jxb/eraf296

This article comments on:

**Renoult SA, Leong JV, Mezzomo P, Sebek P, Koutecký P, Kukla J, Nguyen P, Moos M, Pokorny V, Wagner ND, Frouz J, van Dam NM, Volf M**. 2025. Effects of hybridization on chemical diversity and plant–insect herbivore interactions in *Salix alba×fragilis*. Journal of Experimental Botany **76**, 6974–6986. https://doi.org/10.1093/jxb/eraf296

**In plants, hybridization frequently modifies metabolomic composition in ways that affect ecological performance. According to recent research on *Salix alba × fragilis*, hybrids show intermediate chemical richness but significantly higher structural metabolite diversity, especially in defensive phenolics, which suggests minor structural changes rather than drastic biochemical changes (Renoult *et al*., 2025). These structural subtleties provide insight into how hybrid willows acquire ecological resilience and breadth by mediating interactions with herbivores and soil nutrients.**

Hybridization is a common evolutionary process among plants, contributing significantly to trait diversification and ecological adaptation. Global surveys estimate that hybridization occurs in ∼40% of plant families and 16% of genera (Whitney *et al*., 2010). This phenomenon allows hybrids to occupy new ecological niches and, in some cases, achieve regional dominance. In woody ecosystems such as forests and shrublands, hybrids often constitute a considerable proportion of the vegetation, influencing community interactions, herbivore distributions, and ecosystem functioning (Li *et al*., 2022).

Modern metabolomics offers powerful tools to investigate how hybridization alters specialized metabolites in both quantity and composition. These chemical traits, measured in terms of concentration, structural α-diversity (richness), and β-diversity (between-individual variation), provide insights into how metabolite diversity shapes ecological outcomes (Walker *et al*., 2022). Because hybrid metabolomes can be intermediate or novel, their ecological effects are context dependent. Novel chemical structures may confer broader stress tolerance or reduced herbivore overlap, whereas intermediate profiles can attract herbivores specialized on both parent taxa. Therefore, linking multiple axes of chemical diversity with herbivore specialization and soil variables enables a holistic understanding of hybrid ecological performance.

## Integrating morphological, molecular, and chemical analyses

Renoult *et al*. (2025) investigated how hybridization between *Salix alba* and *Salix fragilis* reshapes phytochemical diversity and mediates ecological interactions with herbivorous insects and adaptability to soil conditions. The study aimed to determine whether hybrids exhibit intermediate, novel, or structurally distinct metabolomic profiles relative to their parental species, and how these chemical patterns influence biotic and abiotic responses. To address these questions, sampling was conducted across a transect of 17 sites in the Elbe Basin, Czechia (Central Europe), representing a range of elevations (100–456 m above sea level) and habitat types from riparian zones to meadows. This spatially distributed sampling captures ecological and geographical variability crucial for studying parental and hybrid species. Morphological identification based on leaf, twig, and flower traits following established taxonomic keys (Gramlich *et al*., 2016) provided an effective first-level classification of *S. alba*, *S. fragilis*, and their hybrids. To minimize repeated sampling of the same genet, morphologically similar stems were sampled only when they were at least 20 m apart. However, as *Salix* species can form extensive clonal structures, genotyping or fine-scale spatial mapping remains necessary for accurate clone delineation (Douhovnikoff *et al*., 2010).

The study combined genomic, chemical, and ecological methods to understand how willow hybrids differ from their parent species. Low-coverage DNA sequencing was used to distinguish species and detect hybrids, complementing morphological classification with reproducible genomic markers (Vašut *et al*., 2024). Leaf chemistry was profiled using high-resolution MS (HRMS), enabling comparison of metabolite diversity across plants (Dührkop *et al*., 2015; Schmid *et al*., 2023). Insects were collected using standard field techniques, and leaf damage was quantified from digital images (Abràmoff *et al*., 2004; Ratnasingham and Hebert, 2013). Soil samples were analysed for key nutrients to relate chemical variation to soil conditions. The data were analysed using statistical models that accounted for differences among field sites and the proportional nature of some measurements (Bates *et al*., 2015; Brooks *et al*., 2017). Patterns in the chemical and ecological data were explored using ordination methods that summarize complex datasets while controlling for site effects (Šmilauer and Lepš, 2014). Finally, metabolite relationships were visualized as networks to show how compounds cluster together (Shannon *et al*., 2003).

## Recombinational metabolomic restructuring drives ecological differentiation in willow hybrids

Genomic profiling based on species-specific repetitive elements diagnostic of *S. alba* and *S. fragilis* classified 34 trees as *S. alba*, 39 as *S. fragilis*, and 52 as hybrids (Renoult *et al*., 2025). Untargeted UHPLC-HRMS metabolomics detected 1081 metabolites, including 84 flavonoids and 65 salicinoids, enabling decomposition of phytochemical diversity into concentration, richness, uniqueness, and structural α- and β-diversity. Hybrids were generally intermediate to parental species in concentration and richness, yet consistently exhibited elevated structural α-diversity across metabolite classes and formed distinct clusters in structural β-diversity analyses for total metabolites and salicinoids. Notably, 95 metabolites were exclusive to hybrids, although most were structurally related to parental compounds, suggesting minor pathway modification and combinatorial restructuring rather than *de novo* biosynthetic innovation.

Herbivore assemblages were structured by metabolite richness and structural α-diversity, with flavonoids influencing beetle communities and salicinoid richness shaping galling insects. However, overall herbivory damage did not differ between hybrids and parental species, suggesting that species-specific positive and negative responses among insect taxa may offset net effects. Soil nitrogen emerged as a minor but significant predictor of structural β-diversity, with hybrids exhibiting greater metabolic variation along nitrogen gradients, particularly in flavonoids associated with both anti-herbivore defence and abiotic stress signalling.

Metabolomic distinction between parental species and hybrids was supported by multivariate ordination studies, with structural composition accounting for a greater proportion of variance than absolute metabolite concentrations. It is noteworthy that the study found little evidence of hybrids massively overproducing metabolites compared with parents; rather, hybrids showed recombinational novelty via minor structural changes within well-established salicinoid and flavonoid scaffolds. Furthermore, network-level analysis revealed that, rather than increasing the overall insect load, chemical restructuring changed the relative abundance of specialist versus generalist herbivores, which in turn affected community assembly patterns.

In hybrid zone characterization, morphological characteristics correlated somewhat with genomic assignment, confirming the reliability of repeat-based genomic diagnoses. The authors conclude that, without triggering completely new metabolic pathways, hybrid metabolomes function by modularly reassembling conserved biosynthetic frameworks to produce chemically detailed phenotypes that control ecological filtering and interaction rewiring. The idea that hybrid zones serve as dynamic evolutionary laboratories where small amounts of biochemical diversity result in quantifiable ecological differentiation and adaptive potential is reinforced by this mechanistic explanation.

Overall, this integrative framework, combining morphological, genomic, metabolomic, and ecological data, demonstrates that hybridization in *Salix alba × fragilis* reconfigures metabolomic architecture by enhancing structural chemical diversity rather than merely increasing metabolite abundance. These findings refine prevailing models of hybrid chemical novelty by showing that moderate structural modifications can reshape multi-trophic interactions while simultaneously enhancing environmental plasticity. In dynamic riparian systems, such metabolomic flexibility may expand niche breadth and contribute to the ecological success and resilience of hybrid zones under changing climatic and edaphic conditions.
